# Interventions to treat mental disorders during pregnancy: A systematic review and multiple treatment meta-analysis

**DOI:** 10.1371/journal.pone.0173397

**Published:** 2017-03-30

**Authors:** Leontien M. van Ravesteyn, Mijke P. Lambregtse - van den Berg, Witte J. G. Hoogendijk, Astrid M. Kamperman

**Affiliations:** 1 Department of Psychiatry, Erasmus University Medical Center, Rotterdam, the Netherlands; 2 Department of Child and Adolescent Psychiatry, Erasmus University Medical Center, Rotterdam, the Netherlands; Chiba Daigaku, JAPAN

## Abstract

**Background:**

For women suffering from an antepartum mental disorder (AMD), there is lack of evidence-based treatment algorithms due to the complicated risk-benefit analysis for both mother and unborn child. We aimed to provide a comprehensive overview of pharmacological and non-pharmacological interventions to treat AMD and performed a meta-analysis of the estimated treatment effect on the psychiatric symptoms during pregnancy.

**Methods:**

MedLine, PsycINFO and Embase databases were searched by two independent reviewers for clinical trials with a control condition on treatment of women with AMD, i.e. major depressive (MDD), anxiety, psychotic, eating, somatoform and personality disorders. We inventoried the effect of the treatment, i.e. decrease of psychiatric symptoms at the end of the treatment or postpartum. We adhered to the PRISMA-protocol.

**Findings:**

Twenty-nine trials were found involving 2779 patients. Trials studied patients with depressive disorders (k = 28), and anxiety disorders (k = 1). No pharmacological trials were detected. A form of psychotherapy, like Cognitive Behavioural Therapy (*g* = -0.61; 95%CI:-0.73 to -0.49, I^2^ = 0%; k = 7) or Interpersonal Psychotherapy (*g* = -0.67; 95%CI:-1.27 to -0.07; I^2^ = 79%; k = 4), holds robust benefit for pregnant women with MDD. Body-oriented interventions (*g* = -0.43; 95%CI:-0.61 to -0.25; I^2^ = 17%; k = 7) and acupuncture (*g* = -0.43; 95%CI:-0.80 to -0.06; I^2^ = 0%; k = 2) showed medium sized reduction of depressive symptoms. Bright light therapy (*g* = -0.59; 95%CI:-1.25 to 0.06; I^2^ = 0%; k = 2), and food supplements (*g* = -0.51; 95%CI:-1.02 to 0.01; I^2^ = 20%; k = 3) did not show significant treatment effects. One study was found on Integrative Collaborative Care.

**Conclusions:**

This meta-analysis found a robust moderate treatment effect of CBT for MDD during pregnancy, and to a lesser extent for IPT. As an alternative, positive results were found for body-oriented interventions and acupuncture. No evidence was found for bright light therapy and food supplements. Only non-pharmacological trials on women with MDD were found. Research on a wider range of AMD is needed.

## Introduction

Antepartum mental disorders (AMDs) are a major cause of disability among women during the perinatal period, and may have consequences for children’s (intra-uterine) growth and development [1, 2]. To date, most reviews and treatments for antepartum mental disorders focussed on depression [3, 4] while a broader range of mental disorders is prevalent during pregnancy and psychiatric symptoms may overlap. The heterogeneity of patients is reflected by estimates from the National Epidemiologic Survey among 43094 American women, showing that the 12-month prevalence of the full range of AMDs did not differ from outside of pregnancy [5], nor resulting in lower rates [6, 7]. According to DSM-IV criteria, most prevalent AMDs were major depressive disorder (MDD), anxiety disorder and psychotic disorder in pregnant women. Prevalence rates of a mental disorder during pregnancy was 25.3%; almost equivalent among postpartum women (27.5%) and non-pregnant women (30.1%) [5].

In a hospital setting, the prevalence of AMDs is similar to cohort studies and additionally a high number of co-morbid mental disorders is found [8–11], e.g. several studies showed that 24.0% had ≥2 or more co-morbid disorders and 5.0% had ≥3 or more co-morbid mental disorders. To clinicians, a pregnant woman can present with a range of psychiatric and somatic symptoms, which sometimes overlap with typical “pregnancy complaints”. For diagnostic and therapeutic purposes, it is important to verify whether a patient dysfunctions in all life domains and to determine the mental disorder(s) according to the DSM-IV criteria. For MDD, DSM-V criteria remained the same and our outcomes can be extrapolated to the current situation. In consultation with the patient, a tailored treatment should be promptly offered, because of the on-going adverse influence of AMDs on the gestation and the increased risk to harmful health behaviours of mother, e.g. smoking, substance use, poor nutrition and avoidance of obstetric care [12, 13]. It has been hypothesized that most relevant effects of AMDs on the foetus take place during mid-gestation and are associated with adverse obstetric outcomes, including preterm delivery, low birth weight, hypertension and preeclampsia [14–17]. To protect the foetus, it is necessary to weigh the potential benefit of treating the mother’s AMD with psychotropic medication against the adverse effects of not treating or relapsing of AMD. There are no suitable data available to guide evidence-based decisions on pharmacological treatment of AMD during pregnancy. Selective serotonin reuptake inhibitors (SSRIs) are the most frequently used pharmacological treatment in pregnant women with MDD, with an estimated 2–3% of women in Europe. There a no studies on (dis)continuation of SSRIs during pregnancy, only two naturalistic studies investigated the preventive effect of SSRI’s for MDD during pregnancy and the results are equivocal [18, 19]. It poses pregnant women and clinicians for a dilemma, what is best for foetus and mother?

From a patient and clinician perspective, there is need to explore evidence for pharmacotherapy and also alternative non-pharmacological treatments for AMDs. In case of depressive disorders, several alternative treatment algorithms in non-pregnant women are shown to be effective [20] and Dennis et al. systematically reviewed these interventions in antenatal depression [21–23]. The authors concluded that for antenatal depression the evidence is too inconclusive to make any recommendations for depression-specific acupuncture, maternal massage, bright light therapy, and omega-3 fatty acids [21]. Various treatments for depression during pregnancy have been systematically reviewed [24–34], however, it remains unclear which non-pharmacological treatment clinicians should offer to pregnant patients with (co-morbid) mental disorders other than depression.

For clinicians it is important to know all available alternative treatments, next to pharmacotherapy, that he/she can offer to a patient with AMD. Our systematic review aimed to provide an overview of randomized or open intervention trials with a control condition that evaluated pharmacological and all non-pharmacological interventions for AMD. Subsequently, the aim of our meta-analysis is to provide an estimation of the overall effect size of a decrease of psychiatric symptoms at the end of treatment or postpartum, for each categorized intervention per mental disorder.

## Materials and methods

### Eligibility criteria

To be selected for inclusion for our review, a trial was required to meet the following criteria:

#### 1. Type of participants

We considered trials that studied pregnant women with a diagnosed mental disorder, with a focus on the following mental disorders and grouped in 7 categories: 1) depressive disorder (MDD, dysthymic disorder); 2) anxiety disorder, e.g. agoraphobia, obsessive-compulsive disorder, panic disorder, phobic disorder, stress disorder, posttraumatic stress disorder; 3) eating disorder, e.g. anorexia nervosa, binge-eating, bulimia nervosa; 4) adjustment disorder; 5) somatoform disorder; 6) schizophrenia and other disorders with psychotics features, e.g. bipolar disorder, or 7) personality disorder. We decided to exclude addiction or any substance-use related disorders, e.g. nicotine-addicts or heroin users. Studies focussing on a population with psychosocial risk factors but without a diagnosed mental disorder were excluded. A prerequisite was that the AMD was diagnosed by means of a (semi-structured) psychiatric interview during pregnancy, e.g. SCID, Mini International Neuropsychiatric Interview (MINI), Composite International Diagnostic Interview (CIDI), Clinical Interview Schedule-Revised (CIS-R) for ICD-10 criteria or Diagnostic Interview Schedule (DIS), and not using screening instruments.

#### 2. Type of treatment

We considered all available pharmacological treatments for AMD, including antidepressants, mood stabilizers, antipsychotics, anxiolytics/tranquilizers and neuroleptics. Also all non-pharmacological interventions for the treatment of AMD were taken into account, including all psychological, body-oriented therapies or other alternative forms of treatment, or combination of these interventions. We included trials that evaluated interventions, which had the primary aim to treat the mental disorder present during pregnancy. Interventions with the focus to prevent–or to treat risk factors for–postpartum psychopathology were excluded.

#### 3. Type of outcome measures

We included all trials that were performed during pregnancy and evaluated the effect of the intervention at the end of the treatment period or closest to delivery in the postpartum period. We inventoried the effect of the treatment on the mental disorder of the mother, i.e. decrease of psychiatric symptoms at the end of treatment or postpartum closest to the delivery.

#### 4. Types of trials

We included all studies with a randomized-controlled (RCT) or open trial design and were published in a peer-reviewed journal. For reasons of validity and quality, we decided to focus only on trials with a control condition and excluded abstracts, case-series and case reports. No language, publication date or publication status restrictions were imposed.

### Search strategy, data abstraction and synthesis for systematic review

**Fig 1:**
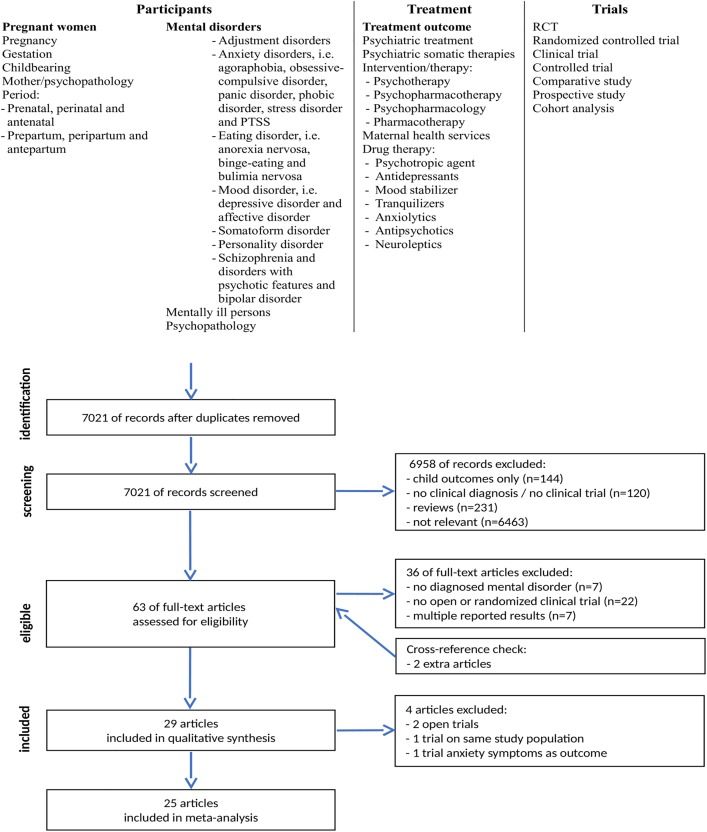
Flow diagram of the review selection procedure, adhered to the PRISMA statement.

Trials were identified by searching electronic databases, scanning reference lists of articles and consultations with experts in the field. MedLine, PsychInfo and Embase were searched from their inception to June 2016 using combinations of the following terms: *Pregnancy*, *Mental disorders*, *Treatment* (see Fig 1 for details and flowchart). To identify other published or unpublished trials, Clinical Trial Databases were searched (clinicaltrial.gov, The ICTRP Search Portal). The last search was run on June 2^th^ 2016. Two reviewers (LR and AK) independently screened all titles and abstracts, excluded protocols and reviews and assessed full-text articles for eligibility. Disagreement between reviewers was resolved by an independent psychiatrist (MLvdB). Results are reported according to the PRISMA-protocol [35], but there was no review protocol (see S4 Table). We assessed inter-rater agreement by kappa statistic using GraphPad Software. A kappa value of 0.61–0.80 reflects substantial, and a kappa-value of 0.81–1.00 (almost) perfect agreement [36]. Details related to the design of the trial, the participants (mental disorder definition and sample size), the description of the treatment and control condition and the outcomes of the trial were extracted from the articles and reported in S1 Table. Most trials used multiple outcomes measures and our interest was to include the outcome measure that operationalized the clinical psychiatric symptoms best, i.e. preference for the Edinburgh (Postnatal) Depression Scale (EDS) which is validated for the use of assessment of depressive symptoms during pregnancy [37]. Our preference was to use the outcome data from the Intention To Treat analysis (ITT), and otherwise data from the per protocol analysis. In case the trial design, procedure or mental health outcomes were reported in an unusual or inconclusive format, the corresponding author of the trial was requested for additional information. Five corresponding authors were contacted and four authors gave the additional information. In situations when multiple articles were drawn from the same trial, the most complete dataset was reported in detail. In case of multiple interventions, both interventions were reviewed, but in case of multiple control conditions only the primary control condition was reported. Pilots or feasibility trials are only mentioned briefly. Subsequently, per subgroup of mental disorders (as described above) the studied interventions and their effect on the mental disorder at the end of therapy or trial, preferably the time point closest to the delivery is presented.

### Risk of bias in individual trials and across trials

The reviewers independently rated the risk of bias for each trial according to the Cochrane Risk of Bias Tool and reported randomization procedure, allocation concealment, blinding procedures and selective reporting in S3 Table [38, 39]. Publication bias was visually assessed with a funnel plot and formally with Egger’s test, to see if the effect decreased with increasing sample size [40]. These plots should be shaped like a funnel if no publication bias is present. However, since smaller or non-significant trials are less likely to be published, trials in the bottom left-hand corner of the plot are often omitted.

### Procedure for meta-analysis

For our meta-analysis, we used the same search strategy as mentioned before and included only randomized controlled trial designs. We excluded open trials. We calculated pooled estimates using bias corrected standardized mean estimates, i.e. Hedges’ *g*, with 95% confidence intervals between the intervention group and the control group at the end of the trial or postpartum closest to the delivery. Hedges’ *g* corrects for the differences in variances resulting from the inclusion of trials with varying sample sizes [41]. The magnitude of Hedges’ *g* can be interpreted as small (0.20), moderate (0.50), or large (0.80) in line with Cohen’s *d* [42]. Pooling was performed per type of intervention and per category of mental disorder over a minimum of two trials. Results for each subgroup of intervention are plotted in a forest plot. Random-effects analysis were used to estimate an overall treatment effect since it produces a more reliable estimate than fixed effect analysis in case of substantial heterogeneity. Cochran’s Q-test, I^2^, and T^2^ statistics were used to quantify heterogeneity across trials. I^2^ >40% was considered as substantial heterogeneity. Heterogeneity was further explored by conducting sensitivity analyses. For this aim, we calculated the overall treatment effect using both fixed and random effects modelling and evaluated the impact of the modelling procedure on the overall treatment effect [43]. Additionally, we created subgroups of trials based on 1) modus of intervention (group-based vs. individual therapy), 2) timing of outcome assessment (end of therapy vs. in the postpartum period), 3) randomization, i.e. secure vs. unknown, 4) allocation concealment (secure vs. unknown/insecure), 5) attrition (less vs. more than 20%), 6) overall study quality (unbiased, unknown/partially biased vs. biased), and 7) outcome measure (questionnaire used), and we evaluated the impact of these moderator variables on the overall treatment effect. Finally, we assessed the influence of the age of the patient as a continuous variable on treatment effect using random effects meta-regression analysis. Standardized effect sizes were calculated using Comprehensive Meta-Analysis (CMA) [44]. Further statistical analyses were performed using the “Metan package” in Stata 13 [45, 46].

## Results

Using our search strategy, we identified 7021 articles (see Fig 1 for a flow diagram). After reviewing the title and abstract, 63 articles were assessed for eligibility and 37 articles did not meet the inclusion criteria. We included 27 articles reporting a clinical trial with a control condition evaluating a treatment for AMD. After cross checking the references, we added 2 relevant articles (A10, A18), thus we included 29 articles in our systematic review. Inter-rater reliability was very good (raw inter-rater agreement = 94%; κ = 0.87). All 29 articles were published in English between 1997 and 2015 (see S1 Table for a summary of all included articles). A reference list of all included articles is presented in S2 Table. Together the articles described 28 unique studies; Burns et al. (A2) and Pearson et al. (A21) published on the same study cohort. Collectively there were a total of 2779 participants in the trials. Almost all participants were diagnosed with a depressive disorder (k = 28) and, to a lesser extent diagnosed with an anxiety disorder (k = 1). No trials were detected with participants diagnosed with AMDs, psychotic disorder, eating disorder, somatoform disorder or personality disorders. Included trials described the effects of a variety of different interventions, e.g. Cognitive Behavioural Therapy (CBT), Inter Personal Therapy (IPT), bright light therapy, body-oriented interventions, acupuncture, food supplements and Integrative Collaborative Care (ICC). The treatment period ranged from 2 to 16 weeks and number of sessions varied between 2 to 32 sessions. Assessment of outcome differed in timing, e.g. end of treatment period (k = 25) or postpartum period (k = 4), and type of questionnaire frequently used were EPDS (k = 9), CES-D (k = 7) and BDI (k = 4). The majority of the trials randomly allocated participants to an intervention or control condition (k = 27), except for two trials with an open design. In the following paragraphs, the results of the different interventions per diagnostic subgroup are described.

### Depressive disorder

In general, the results from 28 unique trials focusing on the treatment of a depressive disorder in pregnant women indicated beneficial effects in relation to a decrease of depressive symptoms at the end of treatment or in the postpartum period. These trials included participants that fulfilled the criteria for MDD according to DSM-IV or ICD-10 criteria. Participants were diagnosed with the SCID (k = 16), MINI (k = 3), DIS (k = 2), CIS-R (k = 2) or other clinical (semi-structured) psychiatric interview (k = 5), often combined with a screening instrument. The majority of the studies were conducted in a Western country (Australia, Sweden, Switzerland, UK, USA) and three trials were conducted in a low-resource country (Pakistan, Korea and Taiwan). The majority of the sample sizes of the included trials were small, varying from 10 to 903 participants and covered in total 2703 participants. Except for one open trial, all trials were (partly) randomized controlled trials.

In total, 8 trials evaluated CBT, 4 trials evaluated IPT, 3 trials examined the use of bright light therapy, 7 trials were on body-oriented therapies, 2 trials on acupuncture, 3 trials on food supplements and one trial on ICC. Participants were individually exposed to the intervention (k = 21) or the treatment was delivered to a group (k = 7). Reduction of depressive symptoms was expressed in scores on the EPDS (k = 9), CES-D (k = 7), BDI (k = 4), HDRS (k = 3), SIGH-SAD (k = 3), CIS-R (k = 2), SCL-20 (k = 1) at the end of treatment (k = 23) or postpartum (k = 3).

#### Cognitive behavioural therapy (CBT)

Austin et al. conducted one of the first large RCTs to demonstrate the superiority of CBT over a booklet and provided weekly 2-hour CBT group sessions for 6 weeks (A1). Per protocol analyses showed that both groups symptomatically improved over time but there was no difference between the two groups. Cho et al. conducted a pilot randomized controlled trial to compare CBT with psycho-education with twenty-seven depressed patients. The intervention group received 9 sessions of individual CBT and had significantly lower rates of depression one month after childbirth (A3). In a low-resource setting, Rahman et al. conducted a large cluster-randomized controlled trial and trained community health workers to provide a CBT-like intervention at home. Although the primary outcome was infant weight and height at 6 months postpartum, less mothers met criteria for major depression in the intervention group than in the control group (OR 0.22 95%CI: 0.14 to 0.36, p<0.001) (A22). Also in another setting, Hayden studied pregnant women with diabetes and with depression (n = 34) and without depression (n = 68), but CBT had no beneficial effect over supportive counseling for both groups (A14). More recent, Burns et al. and Pearson et al. investigated CBT in a pilot RCT and randomized 36 British women who received up to 12 sessions of individual CBT (A2,A21). At 15 weeks post-randomization (linked to a gestational age of approx. 29 weeks), there were more women in the intervention group who did not met ICD-10 criteria for depression any more than in the control group (68.7% vs. 38.5%) and Pearson et al. suggested that the attentional biases of women might improve after CBT. In a pilot RCT, O’Mahen et al. showed that CBT is also a feasible and acceptable treatment for low-income, racial minority women with MDD, however depression scores did not significant differ between the intervention and treatment as usual group (A19). Milgrom et al. showed promising results of an adapted version of a postnatal CBT program, containing 8 antepartum sessions and also reports infant outcomes at 9 months, however post-treatment the depression scores were not significant better for the intervention group (A29).

#### Interpersonal Psychotherapy (IPT)

Spinelli and colleagues were the first to compare a 16-week IPT intervention with parent education matched in time and intensity (A24)[47]. The majority of women were low-income Spanish speaking immigrants, and patients who received IPT had a significant >50% improvement in their mood symptoms. This trial was replicated in 2013 and showed equal benefits of both interventions (A25). Grote et al. reduced the number of sessions from 16 to 8 (brief-IPT) and still a significant larger proportion (95%) of the women in the intervention group no longer met the criteria for MDD compared to the enhanced usual care (58%) at 3 months postpartum (A13). Field et al. studied IPT in a group of women with dysthymia or major depression and after 12 sessions there was no difference in mood symptoms between the intervention and the control peer-support group (A6).

#### Bright light therapy

Three trials have examined the use of bright light therapy for the treatment of antepartum depression. Oren et al. exposed 16 patients for 3 weeks to active bright light in an ABA-design and SIGH-SAD scores improved by 49% from baseline (A20). Withdrawal of bright light treatment was associated with an increase of depressive symptoms. Epperson and colleagues found no significant benefit of bright light over placebo during a 5-week RCT (A4). However, in the extended 10-week trial, active bright light with 20,000 lux had a significant treatment effect compared to 500-lux dim light (effect size 0.43). Wirz-Justice et al. reported a significant difference on HDRS and SIGH-ADS (MD = -5.00, 95%CI:-10.00 to 0.00) scores comparing active (7000 lux) to placebo (70 lux) light therapy after 5 weeks of treatment (A28).

#### Body-oriented interventions

Field et al. studied extensively alternative antepartum interventions for depression (A5-A11). Massage by a significant other, compared to standard care significantly decreased the number of women with depressive symptomatology on the Center for Epidemiological Studies Depression Scale (CES-D) immediately post-treatment in a small (n = 47, MD = -4.9) (A11) and bigger sample (n = 149, MD = -6.7, 95%CI:-9.8 to -3.6) (A9). Field et al. studied also group-IPT in pregnant women and added 6 sessions of massage therapy for the intervention group (A5). The group that received both interventions, showed a greater decrease in depression and anxiety scores. Recently these authors compared yoga or massage therapy twice weekly (A10), tai chi/yoga therapy (A8) and weekly yoga to standard antepartum care for 12 weeks (A7). These three trials showed a significant greater decrease of depression and anxiety scores in the intervention groups compared to the control groups. Uebelacker compared group yoga with a mom-bay wellness workshop and found no difference in depression scores (A27).

### Acupuncture

Two trials examined the role of acupuncture, Manber and colleagues studied depression-specific acupuncture in comparison with non-specific acupuncture and massage therapy (A16-A17). In a small sample in 2004, there were no differences in pregnant women diagnosed with clinical depression after treatment nor at 10 weeks postpartum (A16). A new sample of 150 patients in 2010 showed that women who received acupuncture specific for depression experienced a greater reduction of HDRS-rates, compared with the combined controls or control acupuncture after 8 weeks of treatment (A17).

### Food supplements

Three trials have explored the potential value of food supplements. For example, Freeman performed a randomized double blind placebo controlled trial to compare the use of omega-3 fatty acids to placebo, with supportive psychotherapy provided to all patients (A12) and studied therapy adherence [48]. Both groups experienced a significant improvement in self-reported and observer rated depression over 8 weeks, although there were no group differences. In contrast, Rees et al. published a negative but properly executed trial on the use of omega-3 fatty acids in a double blind, placebo-controlled trial (A23). Su et al. concluded the superiority of omega-3 fatty acids and showed that the intervention group had significantly lower mean HDRS scores (MD = -4.70, 95%CI:-7.82 to -1.58) after 8 weeks of treatment (A26). At the trial endpoint, patients in the omega-3 group also had lower depressive symptom ratings on the EPDS and BDI.

#### Integrative collaborative care (ICC)

Multidisciplinary care and personalized care have received a lot of attention. Melville et al. evaluated their ICC in a RCT at an obstetric outpatient clinic which included an engagement session, an assessment by a Depression Care manager and potentially supported by antidepressant medication or problem-solving therapy for primary care for 1 to 4 weeks (A18). After one year, the intervention group had significant greater decrease of depressive symptoms on the Hopkins Symptom Checklist-20 compared to the usual care group.

### Anxiety disorders

Only one trial fulfilled the inclusion criteria for the treatment of anxiety disorders in pregnant women. This trial included patients that met criteria for anxiety disorder or blood- and injection phobia according to DSM-IV criteria. The sample size of the included trial was 76 patients and evaluated CBT in an open trial (A15). Patients received two sessions of group-CBT and were compared with 46 women diagnosed with blood- and injection phobia, but untreated. CBT-treated women scored significantly lower after each session and postpartum on anxiety and avoidance scores.

### Methodological quality of trials and risk of bias

Data available for the meta-analysis was provided by 25 trials and methodological quality was reported in a risk of bias table in the appendices. Twelve trials did not describe the procedure of concealment of allocation and randomization. Blinding of the participants was not always feasible, but in twelve trials participants or assessors were blinded. Attrition rates varied from 0 to 52%. Majority (72%) of the trials did not publish a protocol and/or was registered in a trial register. Inter-rater agreement with regards to the quality assessment was substantial (raw inter-rater agreement = 83%; κ = 0.70).

**Fig 2:**
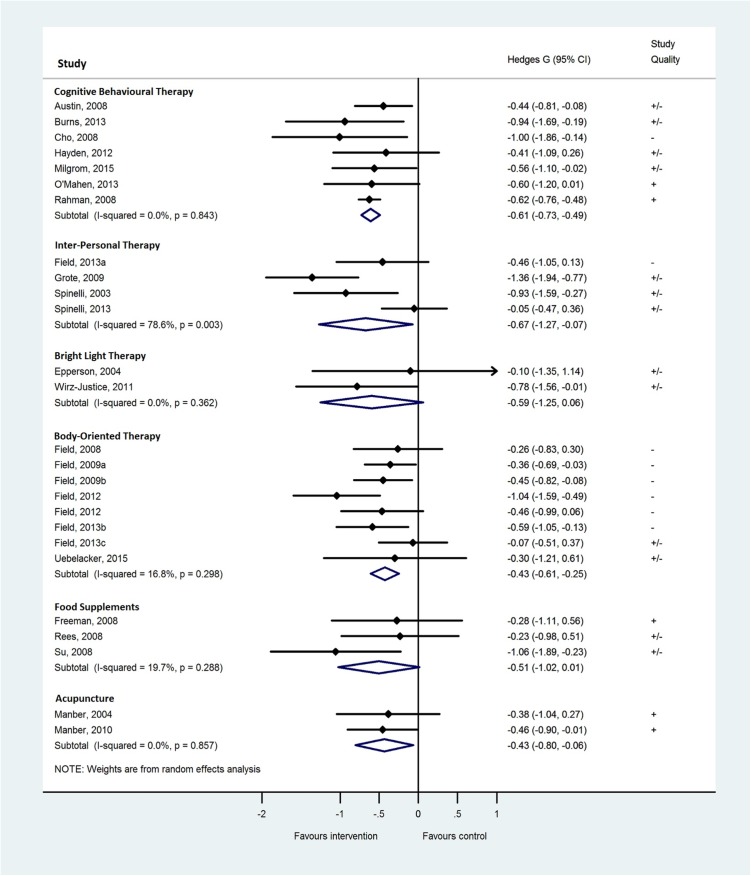
Funnel plot including pseudo 95% confidence limits of the included trials (k = 25) stratified by intervention.

A visual inspection of the funnel plot revealed that the plot was symmetric, so we had no indication of a publication bias (see Fig 2). Only three studies were identified outside the pseudo 95% confidence interval (A10,A12,A24). Also, the Egger’s test did not suggest the presence of publication bias (β = 0.08; 95%CI:-0.83 to 0.99; p = 0.86).

### Meta-analysis

**Fig 3:**
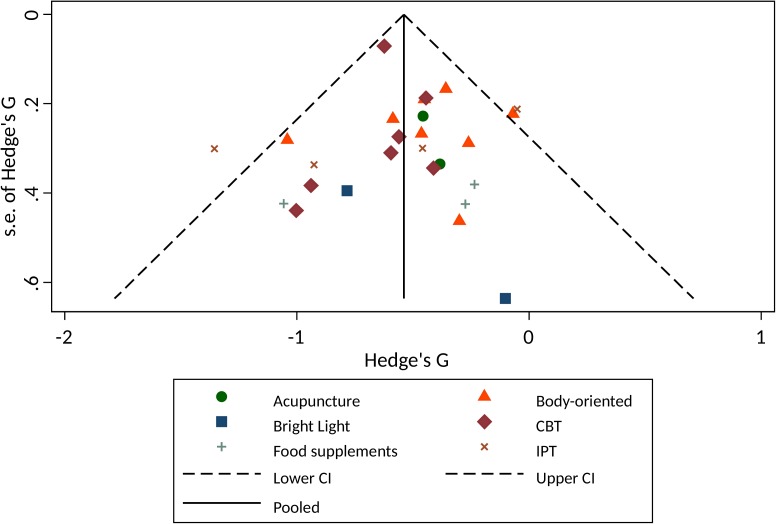
Hedges *g* of interventions to reduce depressive symptoms in pregnant women.

For the patients with depression, we grouped the available interventions together in 1) CBT; 2) IPT; 3) bright light therapy; 4) body-oriented therapies; 5) acupuncture; and 6) food supplements (see Fig 3). No overall statistics were calculated for Integrating Collaborative Care (k = 1) and a trial focussing on patients diagnosed with an anxiety disorder (k = 1). For each intervention subgroup, we compared all available trials on improvement of psychiatric symptoms and study quality as reported in S1 Table. Analysed in a random effect model, the psycho-therapeutically interventions were both associated with reduction of depressive symptoms. In case of CBT, treatment size was medium with little inconsistency between trials (*g* = -0.61; 95%CI:-0.73 to -0.49), and overall effect was significant (Z-value = 10.04; p<0.001). Among the 7 trials evaluating CBT, there was no evidence of heterogeneity (Tau^2^<0.001; Chi^2^(6) = 2.72; p = 0.84; I^2^<1%). In case of IPT the effect was also medium. However, the magnitude of the imprecision shows large inconsistencies between the trials (*g* = -0.67; 95%CI:-1.27 to -0.07). Inconsistency among the four IPT trials was supported by the test for heterogeneity (Tau^2^ = 0.29; Chi^2^(3) = 14.01; p<0.001; I^2^ = 79%). Overall the treatment effect of IPT was significant (Z-value = 2.20; p = 0.03). Overall treatment effect of bright light interventions was not associated with a decrease of depressive symptoms (*g* = -0.59; 95%CI:-1.25 to 0.06; I^2^ = 0%; Z-value = 0.77; p = 0.08). Heterogeneity was not tested significantly (Tau^2^<0.001; Chi^2^(1) = .83; p = 0.36; I^2^<1%). Body-oriented intervention was associated with a medium sized improvement, consistent over the trials (*g* = -0.43; 95%CI:-0.61 to -0.25), overall treatment effect was significant (Z-value = 4.62; p<0.001). Heterogeneity was not tested significantly (Tau^2^ = 0.02; Chi^2^(7) = 8.41; p = 0.30; I^2^ = 17%). Treatment with food supplements was not associated with decrease of depressive symptoms (*g* = -0.51; 95%CI:-1.02 to -0.01; Z-value = 1.92; p = 0.06). Heterogeneity was not tested significantly (Tau^2^ = 0.04; Chi^2^(2) = 2.49; p = 0.29; I^2^ = 20%). Finally, the two trials evaluating acupuncture showed a significant medium overall treatment effect (*g* = -0.43; 95%CI:- 0.80 to 0.07; Z-value = 2.30; p = 0.02). Heterogeneity was not tested significantly (Tau^2^<0.001; Chi^2^(1) = 0.03; p = 0.86; I^2^<1%).

#### Sensitivity analysis

**Fig 4:**
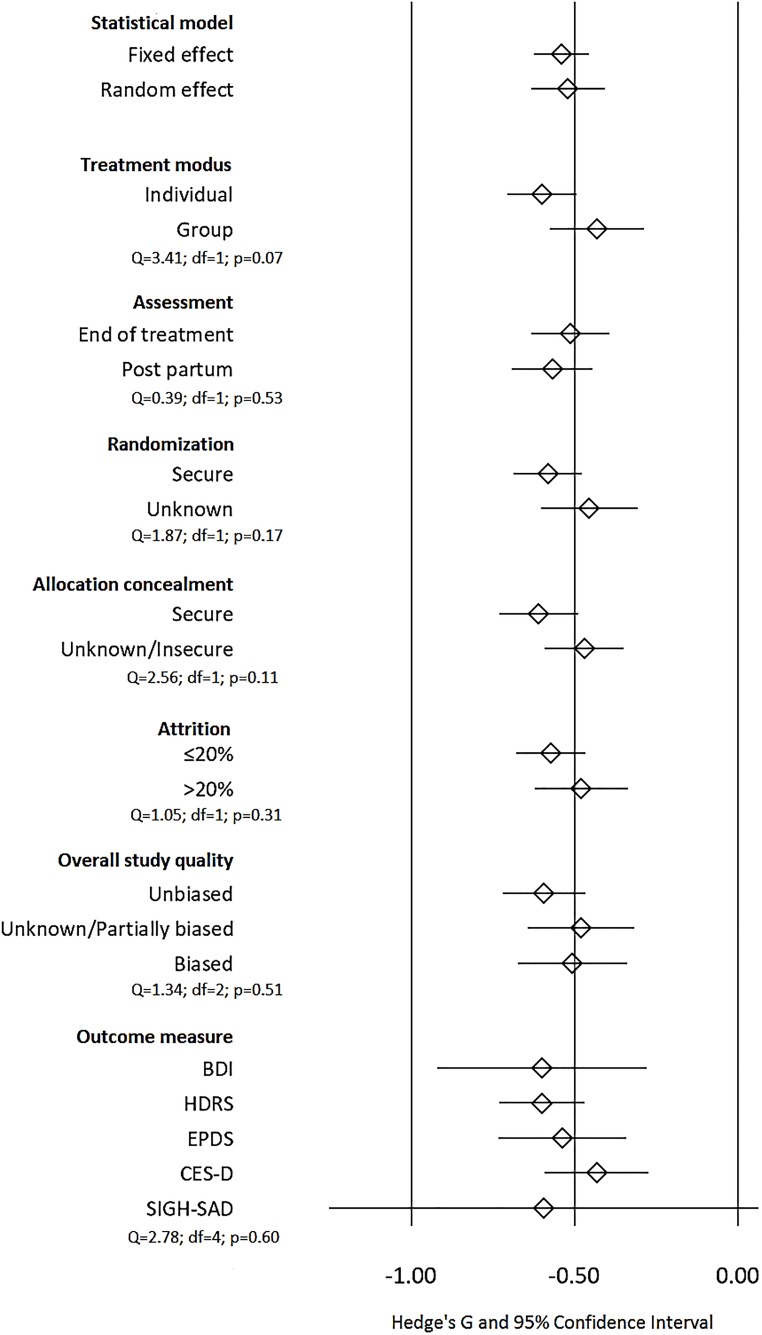
Intervention effect in the full set of included trials (k = 25) using fixed and random estimation, and for different subgroups of trials. Pooled effect sizes for subgroups of trials are estimated using random-effects estimation. Fixed-effect estimation was used to compare differences over subgroups.

Fig 4 depicts the results from the sensitivity analyses. As the figure shows, the overall treatment effect regarding symptoms of depression is robust to intervention and trial characteristics and statistical method. The mode of intervention, i.e. group vs. individual therapy, nor any of the trial design characteristics, i.e. outcome measure, moment of assessment, nor any of the trial quality characteristics showed a significant impact on the overall intervention effect. Regression analysis revealed no significant association between treatment effect and the age of the included patients (β = 0.01; 95% CI:-0.03 to 0.06, p = 0.69).

## Discussion

### Summary of evidence

The aim of this systematic review was to provide an overview of trials that evaluated pharmacological and non-pharmacological interventions for AMD and in addition to provide an estimation of the overall effect size of categorized interventions per mental health outcome. Given the importance of treating mental disorders during pregnancy for mother and child, this meta-analysis extends the literature [29, 33, 34, 49] by thoroughly examine all available treatments for AMD.

Until this date there are no controlled studies on the effect of psychotropic medication for AMD. We could only estimate effect sizes for treatment of patients diagnosed with Major Depressive Disorder (MDD) by a lack of studies on other mental disorders during pregnancy. A form of psychotherapy for MDD has robust effect sizes, e.g. Cognitive Behavioural Therapy (CBT, *g* = -0.61), and to a lesser extent Interpersonal Psychotherapy (IPT, *g* = -0.67). Both may hold potential benefit for pregnant women with MDD in this analysis. This is in line with current NICE guideline that advises clinicians to offer a form of psychotherapy to every pregnant woman with a history of mild to severe depression and emphasizes close consultation with patients [50].

Other potential beneficial non-pharmacological intervention categories to treat MDD were body-oriented interventions and acupuncture. Our data suggests that bright light therapy is not associated with a decrease of depressive symptoms, but this is based on two trials. Overall, we identified only a small number of studies for each intervention category with small sample sizes and potential risks of bias. We performed sensitivity analyses to evaluate the impact of these moderator variables on the overall treatment effect, but none showed to be significant. However, the ability to perform certain moderation analyses was limited by the size and quality of current English literature. In the majority of the trials only per protocol data was available and this has likely resulted in an overestimation of our effect sizes.

Our results showed that the overall effect sizes of all non-pharmacological intervention are in close range to each other and may be redeemable for one other, bearing in mind the high attrition rates of most trials. Furthermore, the effect sizes are similar or even higher than the effect of psychotropic medication in non-pregnant depressed patients [51–53]. In summary, the effect sizes of the different interventions to treat depressive symptoms are close to each other and therefore we suggest that the preference of the patient have to weigh heavily in the decision for a psychiatric treatment in a clinical setting.

### Strengths and limitations

To our knowledge, a broad approach to examine various interventions for mental disorders during pregnancy is not performed before. We limited our study to a clinical group of patients with a diagnosed disorder, in order to extrapolate the evidence on treatment of AMDs for clinicians. The disadvantage of this approach is that we missed potentially effective interventions in a healthy population that could be beneficial also for a clinical sample. By pooling the interventions by six subgroups in our meta-analysis and conducting several sensitivity analyses, we believe that we have been able to show the effects of each intervention and gained insight in sources of variability between the included studies. Our estimates are lower than other meta-analysis which report average effect for IPT, ranging between 1.14 (one-group studies) [33] to 1.26 [29]. We focused only on the treatment period during pregnancy, on a clinical diagnosis and controlled trials, therefore our results show a more robust estimate of the beneficial effect of IPT for pregnant women with MDD. Post-hoc analysis of Claridge et al. focusing on high quality clinical samples, found similar effect sizes (d = 0.40) [33].

We considered also open trials in our qualitative systematic review, because it is known that this hard-to-reach population is difficult to enrol and randomize for trials, due to practical and ethical reasons. As indicated by the funnel plot and Egger test, there was likely no publication bias in this synthesis, and also our sensitivity analysis showed no potential biases. Altogether, the advantage of our broad and systematic approach resulted in a comprehensive overview and robust results.

### Implications and conclusions

This meta-analysis contributes to the literature in several ways. Our review shows that the number of trials on treatment of AMD is low, although rising every year. No controlled studies were found to show evidence for the use of psychotropic medication during pregnancy. We highlight the continuing need for further research of antepartum treatment for the full spectrum of AMDs, e.g. anxiety disorders, psychotic disorders, eating disorders, psychosomatic disorder and comorbidity like personality disorders. The evidence provided is inconclusive, and is predominantly based on trials evaluating major depressive disorder during pregnancy in small sample sizes. It is recommended that future research include other mental disorders in larger numbers and study alternative non-pharmacological interventions in comparison with pharmacotherapy. Findings of alternative interventions offer the promise of efficacy without the complexity of weighing pros and cons regarding foetal exposure to psychotropic medication and maternal stress. For example body-oriented therapies (*g* = -0.43) and acupuncture (*g* = -0.43) are promising alternatives, but the evidence is based on two to four trials and the results should be replicated, preferably by researchers from different institutes. The results of omega-3 fatty acids intake are mixed and also a recent meta-analysis in non-pregnant depressed patients suggests a small, non-significant benefit. However, nearly all of the treatment efficacy might be attributable to publication bias [54]. Bright light therapy showed to be effective for the treatment of non-seasonal depression in non-pregnant population [55], but needs further research in pregnant women.

Our systematic review found also a high number of protocols, which are promising as well. For example protocols for tapering antidepressants during pregnancy [56], for a broader range of mood disorders [57–59] and a rise of mindfulness-based therapies is observed [60]. Due to the small number of participants or weaknesses in design of the studies, we could not include other alternative interventions that showed promising effect in case-series, e.g. Electroconvulsive Therapy [61] and Transcranial Magnetic Stimulation [62, 63], or in healthy pregnant women, e.g. exercise [64], music therapy [65] or multicomponent psychotherapy [66]. For future research it would be interesting to examine the association between the severity of the disorder with the improvement of psychiatric symptoms to further personalize treatments. It should also be noted that the evidence was identified for short-term outcomes of AMD, and that further research is needed to evaluate longer-term mother and child outcomes.

To conclude, in the field of perinatal psychiatry there is a lot of attention for depression and a couple of evidence-based therapies are available and redeemable. However, the broader range of mental disorder are not represented in current literature, while anxiety, bipolar and other psychotic disorders may adversely affect mother and foetus, we strongly recommend further research on both pharmacological and non-pharmacological treatment options for all mental disorders during pregnancy.

## Supporting information

S1 Table Summary of included trials evaluating interventions to improve mental health outcomes during pregnancy. (TIFF)

S2 Table Reference list of all included articles. (TIFF)

S3 Table Risk of bias table of all included trials. (TIFF)

S4 Table PRISMA-checklist. (TIFF)
