# Supplementary figures and images for: Interventions to treat mental disorders during pregnancy: A systematic review and multiple treatment meta-analysis
Source: PLoS One. 2017 Mar 30;12(3):e0173397. doi: 10.1371/journal.pone.0173397 (PMC5373816; doi:10.1371/journal.pone.0173397)

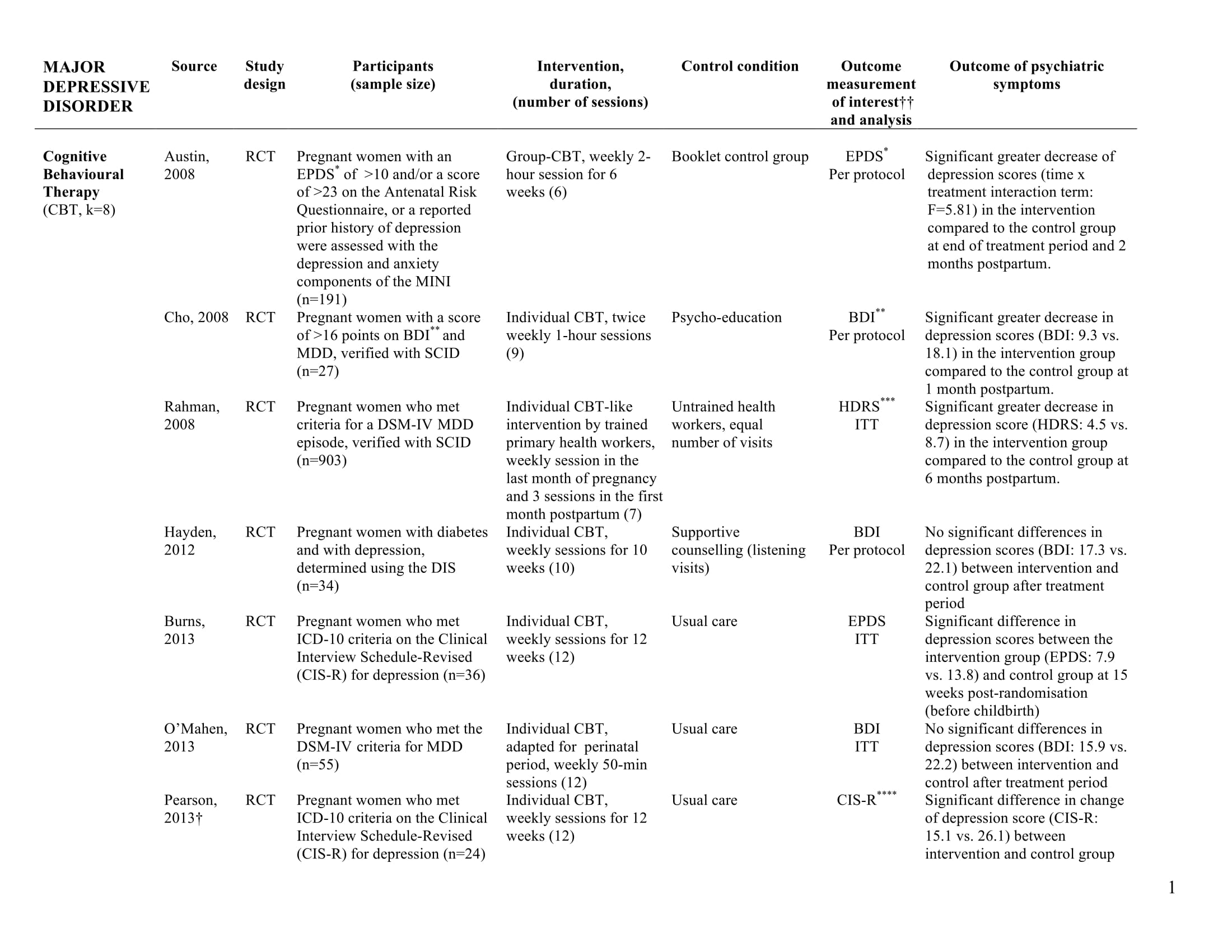

Supplement: S1 Table — (TIFF) [file pone.0173397.s001.tiff]

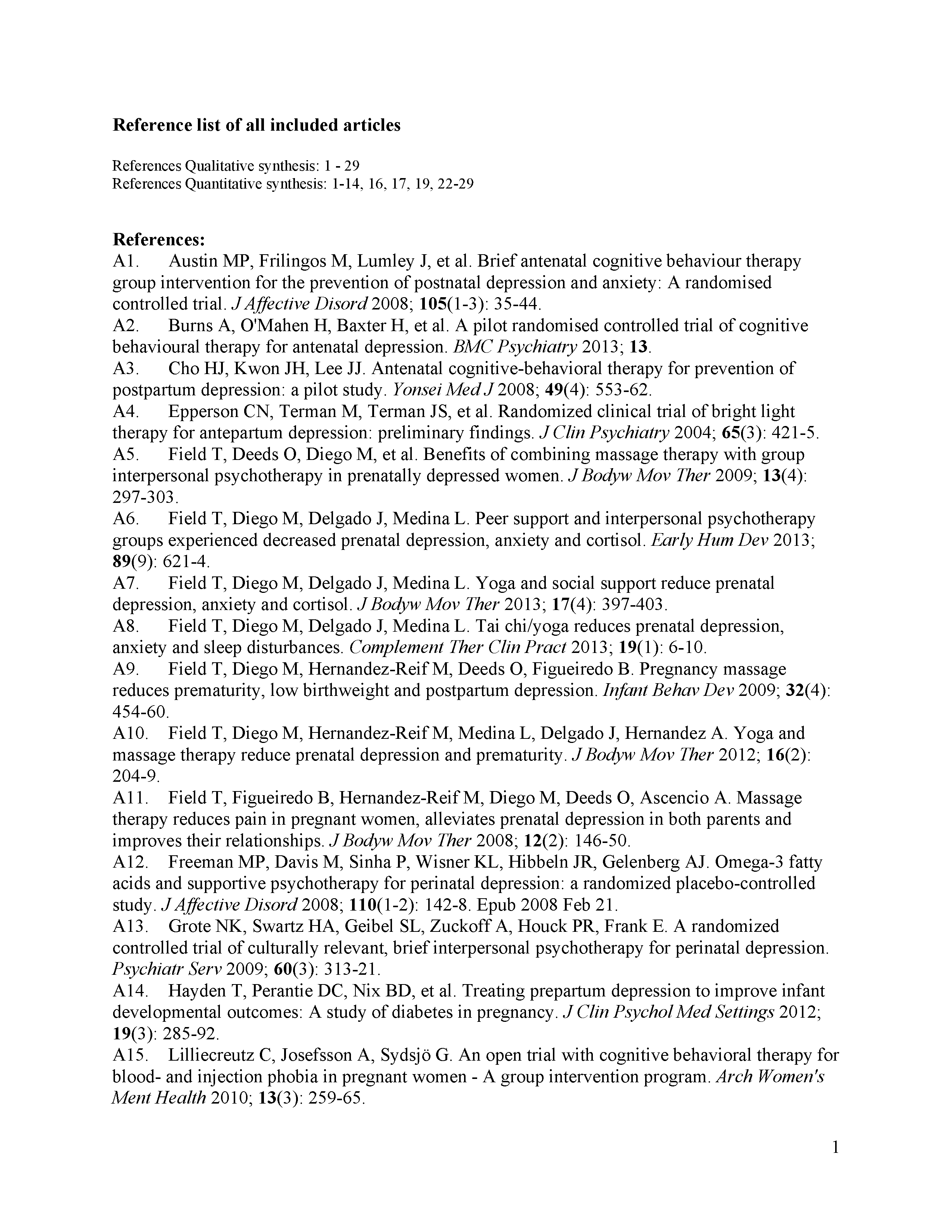

Supplement: S2 Table — (TIFF) [file pone.0173397.s002.tiff]

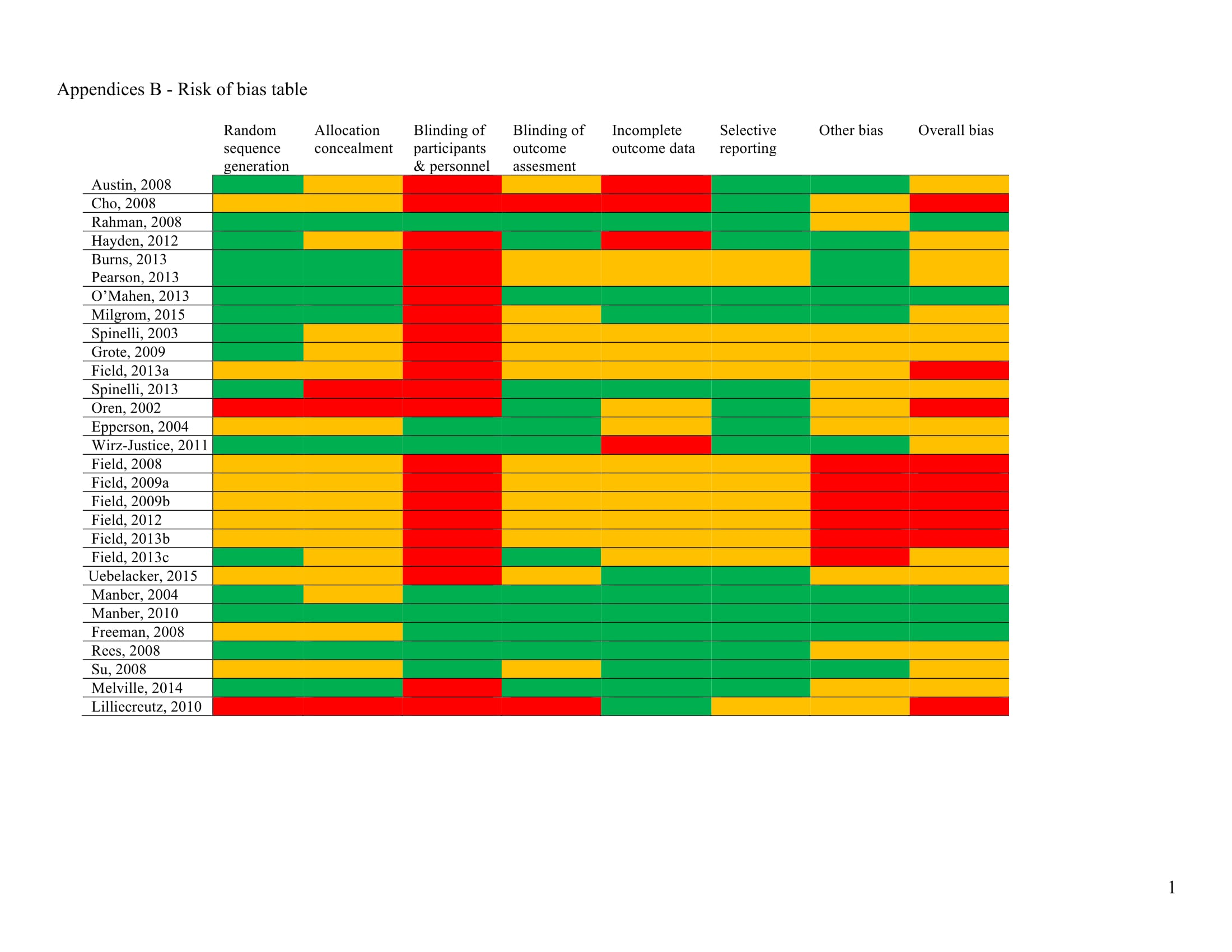

Supplement: S3 Table — (TIFF) [file pone.0173397.s003.tiff]

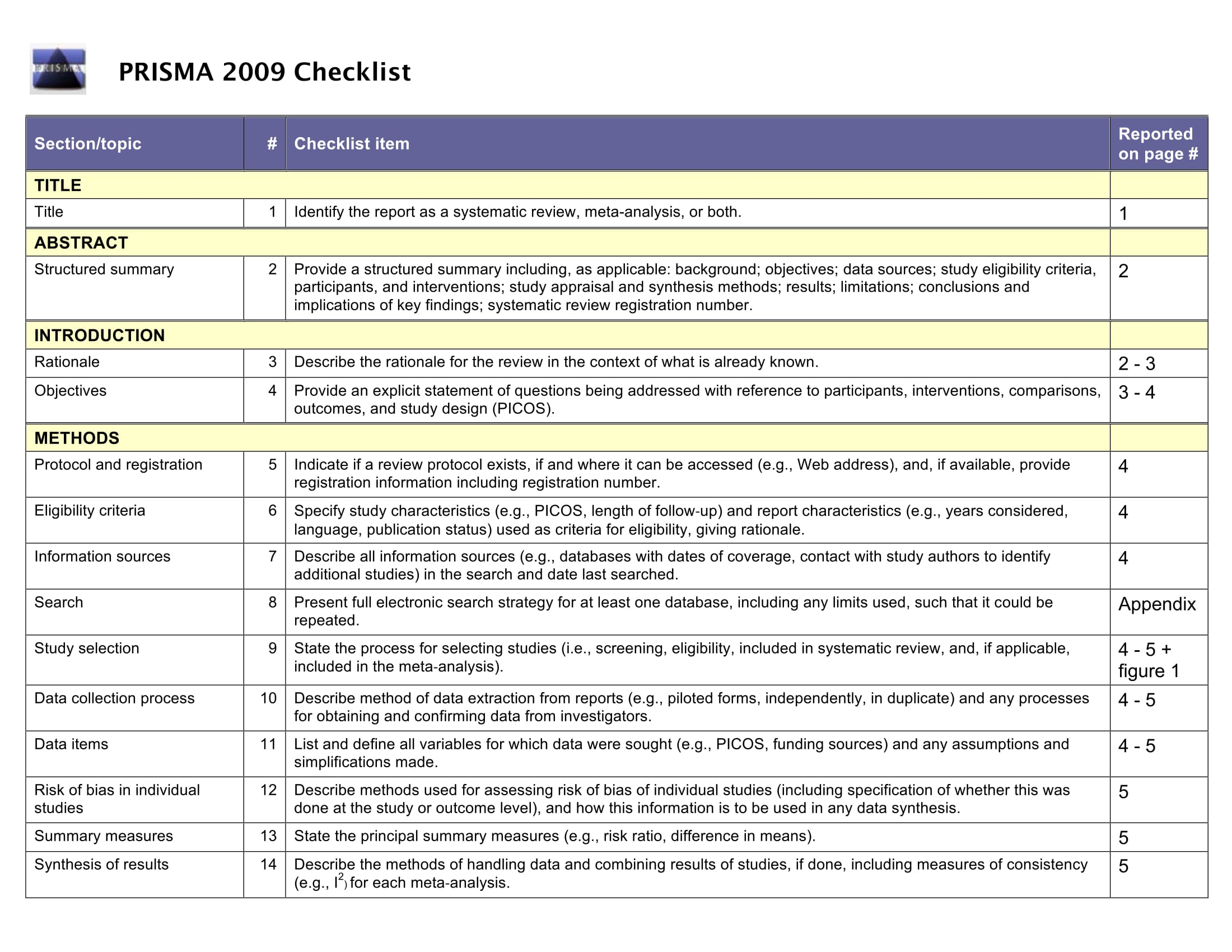

Supplement: S4 Table — (TIFF) [file pone.0173397.s004.tiff]
